# Comparison of the Mechanical Properties of Hardfacings Made by Standard Coated Stick Electrodes and a Newly Developed Rectangular Stick Electrode

**DOI:** 10.3390/ma17092051

**Published:** 2024-04-27

**Authors:** Edvard Bjelajac, Andrej Skumavc, Gorazd Lojen, Mirza Manjgo, Tomaž Vuherer

**Affiliations:** 1 Messer Slovenija d.o.o., Jugova ulica 20, 2342 Ruše, Slovenia; edvard.bjelajac@messergroup.com; 2 SIJ Acroni d.o.o., Cesta Borisa Kidriča 44, 4270 Jesenice, Slovenia; andrej.skumavc@acroni.si; 3 Faculty of Mechanical Engineering, University of Maribor, Smetanova ulica 17, 2000 Maribor, Slovenia; gorazd.lojen@um.si (G.L.); mirza.manjgo@student.um.si (M.M.)

**Keywords:** hardfacing, dilution rate, hardness, Charpy impact toughness, residual stress, fracture toughness

## Abstract

Cladding with a stick electrode is one of the oldest arc processes for adding a deposit on a base material. The process is suitable for outdoor working, but the disadvantages are low productivity and large dilution rates. In this work, a simple solution is proposed, which would enable cladding of a larger area with one pass and decrease the dilution rate at the same time—a new type of electrode was developed, exhibiting a rectangular cross-section instead of a round one. Hardfacings, welded with E Fe8 electrodes according to EN 14 700 Standard were welded on mild steel S355 J2 base material with three different coated stick electrodes. The first one was a commercially available, standard, round hardfacing electrode, the second was the same, but with a thinner coating, and the third one was a newly developed rectangular electrode. All three types had equal cross-sections of the metallic core and the same type of coating. Manufacturing of the rectangular electrodes in the laboratory is explained briefly. One- and multi-layer deposits were welded with all three types. Differences were observed in the arc behavior between the round and rectangular electrodes. With the rectangular electrode, the microstructure of the deposit was finer, penetration was shallower, and dilution rates were lower, while the hardness was higher, residual stresses predominantly compressive, and the results of instrumented Charpy impact tests and fracture mechanics tests were better.

## 1. Introduction

In general, wear is mechanically induced surface damage that results in the progressive removal of material due to relative motion between the surface of a solid body and a contacting substance. The contacting substance can be the surface of another solid body, soil, gravel liquid, or abrasive particles carried by a gas or liquid flow. In terms of safety, wear is often less dangerous than fracture because wear is usually anticipated [1]. Nevertheless, the occurrence of wear is highly undesirable because it leads to the deterioration or even failure of components.

Wear is classified commonly according to the so-called wear types, which are caused either by a single mechanism or by complex interactions of several mechanisms. Usually, interactions of several mechanisms result in higher wear rates than exposure to individual wear mechanisms [1]. The most common types of wear are abrasive wear [2,3,4,5], adhesive wear [6,7,8], surface fatigue [9,10], fretting wear [11,12], erosion wear [13], and corrosion and oxidation wear [14]. Less common types are diffusive wear [15], impact wear [16]**,** and cavitation wear [17].

Hardfacing is a process where a harder and/or tougher material is applied to the surface of a base metal to extend a service life of new components or to repair worn-down surfaces of old components [18].

State-of-the-art hardfacing alloys comprise very cost-efficient Fe-Cr-C and Fe-C-B systems on the one hand, but, on the other hand also more expensive materials are available, e.g., synthetic multiphase composites reinforced with tungsten carbides [19]. However, more expensive tungsten- or vanadium-rich alloys offer better performance due to their good combination of hardness and toughness. Complex carbides are also used, especially when abrasive wear is accompanied by other wear mechanisms [20]. The choice of the optimum hardfacing process depends on the workpiece to be hardfaced, in particular, on the function of the component, its size and shape, base metal composition, location where the work is to be carried out, accessibility of the surface to be cladded, and the number of identical or similar items to be hardfaced.

**Figure 1:**
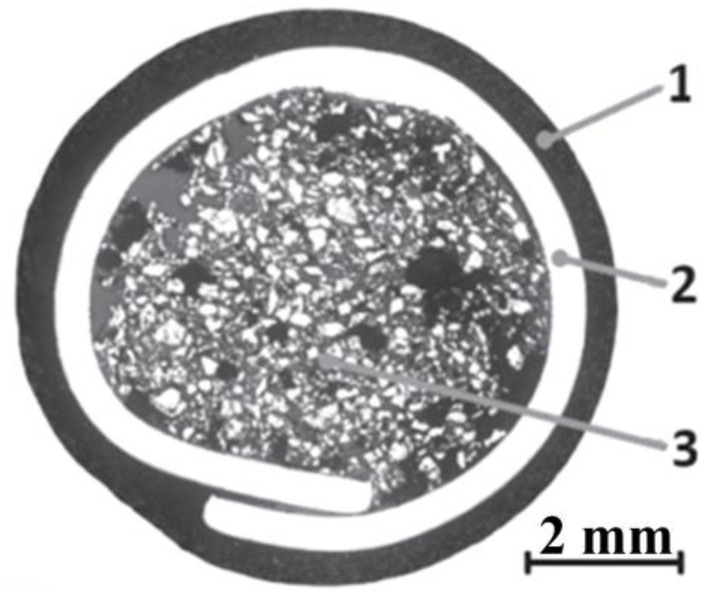
The cross-section of a coated tubular electrode with a seam: 1—coating, 2—metal tube, and 3—powder core [39].

Different processes are used, depending on the desired thickness of the layer to be applied [21]. The deposition of overlay coatings by welding or thermal spray techniques is employed frequently (either during maintenance or for manufacturing new components) in a wide range of industries [22], including agriculture [23,24,25,26], mining [27,28,29], nuclear [30], automotive, chemical, metal working, shipbuilding, and the petroleum industry [31]. The most common processes are oxy-acetylene welding (OAW) [32,33], shielded metal arc welding [34,35], gas metal arc welding with self-shielded or gas shielded flux wires (FCAWs) [36,37], gas metal arc welding (GMAW), plasma transfer arc welding (PTAW) [38], gas tungsten arc welding (GTAW) [31], and submerged arc welding. A conventional electrode for SMAW welding consists of a metal core in the middle and non-metal coating around it. Similar to conventional coated electrodes, tubular coated cored electrodes consist of a thin-walled tube, or a coiled metal strip filled with powder, and a thin non-metallic coating outside (Figure 1). Compared to coated solid core electrodes, tubular electrodes enable higher deposition rates and higher metal recovery, shallower penetration and lower dilution rates at the same deposition rate, lower welding parameters and consequently a lower heat input, no need for storage in special conditions, and drying before use [39]. Furthermore, it is possible to add higher volumes of alloying elements into the core and, hence, higher levels of alloying elements and carbides in the deposit, leaving the outer coating only for the other referred technological properties [40]. Underwater wet welding was developed with tubular coated electrodes [41].

In this study, we present a newly developed rectangular coated electrode. With the application of the rectangular metallic core of the stick coated electrode, we expected to observe a similar influence on the arc droplet formation mechanisms as was reported for tubular electrodes [41]. Moreover, we expected to influence the heat distribution in the base material and decrease the penetration and dilution.

With a lower dilution rate, this type of electrode should reduce the necessary volume of filler material (less layers needed), and, thereby, reduce the welding time, lower the overall costs, and reduce the welder’s exposure to welding gases.

To our knowledge, rectangular electrodes have not been used yet for SMAW. They cannot be found in catalogs of filler material producers, nor have they been reported in the literature. Hence, the development of rectangular electrodes represents a new approach to hardfacing in demanding conditions outside the production halls, and lays the foundation for the further development of and optimization of their cross-section dimensions, coating thicknesses, and chemical compositions, not only for purposes of hardfacing, but also for other applications.

For the purpose of this investigation, we manufactured, in laboratory conditions, rectangular-shaped coated electrodes with a metal core cross-section of 1 mm × 12.56 mm, which corresponds to the core cross-section of a ∅4 mm standard electrode. The results obtained with our rectangular electrodes were compared with the results obtained with standard, round, hardfacing electrodes at ∅4 mm with two different thicknesses of coating. Optical and SEM microscopy tests of the welds were conducted, dilution rates were calculated, and microhardness tests, instrumented Charpy impact toughness tests, fracture mechanic tests were performed, along with residual stresses measurements.

## 2. Experimental Procedure

### 2.1. Production of Rectangular Stick Electrodes

Coated stick electrodes having a rectangular cross-section were developed and manufactured in laboratory conditions. Different ratios of the width/thickness of the rectangular metallic cores’ different coating thicknesses and different welding parameters were tested in the preliminary research. It was found that, with decreasing the width/thickness ratio, rectangular electrodes behaved more and more like round ones, until, at sufficiently low ratios, the arc was not moving any more. On the contrary, with increasing the width/thickness ratio, the distance of arc-traveling increased, while the maximum local heat input, penetration, and dilution rate decreased. The following core cross-sections were tested: 2 × 6.28 mm, 1 × 12.56 mm, 2 × 30 mm, and 1 × 30 mm. To assure the best possible comparison with standard ∅4 mm round electrodes, which were selected for comparison, the same size of cross-section, with the dimension of 1 × 12.56 mm, was selected for this study.

Testing of different combinations of welding parameters revealed that 120 A was the optimum. Some details about these tests are reported in [42,43]. For the ∅4 mm round electrode, a current in the middle of the recommended range [44], 160 A, was found to be a good compromise between undemanding welding and a not too high heat input.

The metal cores measuring 205 × 12.56 × 1 mm were made using a laser cutting machine, AMADA PROMECAM LC 2415 Alpha IV NT (AMADA Ltd., Kidderminster Industrial Estate, Spennells Valley Rd, Kidderminster DY10 1XS, UK), 4 KW from DC01 cold-rolled mild steel according to EN 10130 Standard [45]. The welding equipment’s producer, SIJ Electrode Jesenice d.o.o, Jesenice, Slovenia, donated rutile-type electrode coating, the same mixture used in the production of their standard hardfacing electrodes E DUR 60 R, having a standard designation of E Fe8 according to EN 14 700 [46], with a typical weld deposit hardness of 55–60 HRC.

Since the goal of this investigation was to evaluate the influence of the cross-section shape, possibly with the exclusion of other influences, for the rectangular electrode, identical coating was deliberately selected as that used for commercially available round electrodes; the same steel was used for the metallic cores, the same size of metal core cross-sections, and the same ratio of metal core/coating cross-sections as one of the used round-electrode types.

For manufacturing, constructed aluminum molds and a hydraulic press were used for in study. Water glass was added to the dry powder mixture to obtain a viscous mass. The wet mass was pressed into a flat shape. Then, it was cut into strips. A strip was put into the aluminum mold, a metal core was inserted, and another strip of coating was laid over the core. Finally, the electrode was pressed with a compression force of 60 kN. After pressing, the electrodes were dried in the air for 3 days, followed by drying in a laboratory furnace at 350 °C for 3 h. The final step was removing the excess coating from the edges by grinding.

**Figure 2:**
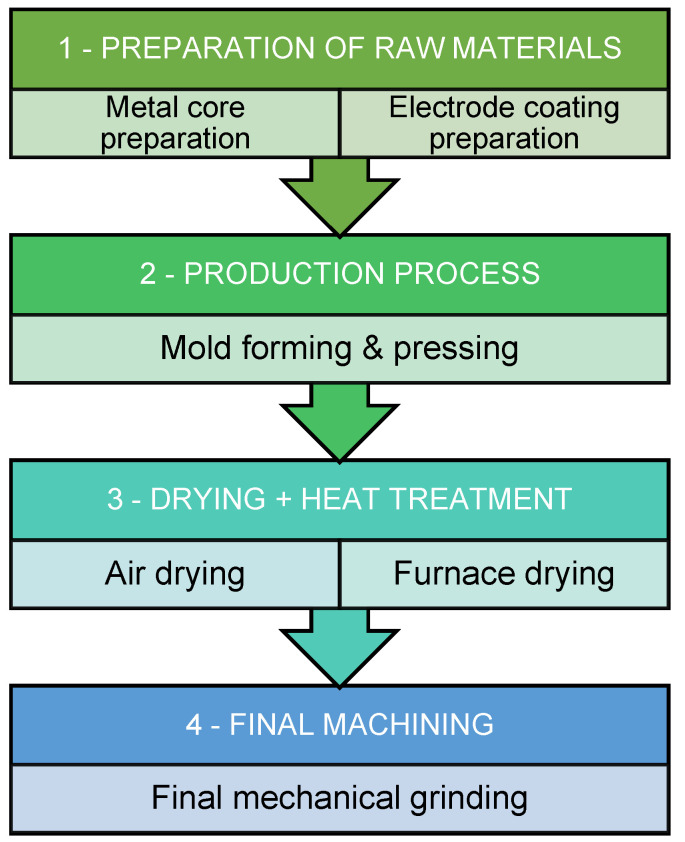
Method of laboratory production for rectangular coated stick electrode.

**Figure 3:**
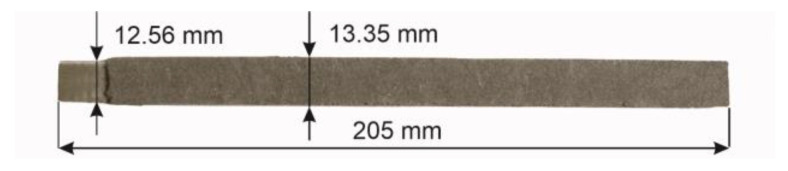
A rectangular coated electrode for SMAW welding.

The scheme of the manufacturing process is shown in Figure 2, and the rectangular electrode in Figure 3.

To improve the welding properties, the coating thickness of the rectangular electrodes was optimized in a series of preliminary tests. In this study, the outer cross-section dimensions were ∅2.80 × 13.35 mm and the core-to-coating cross-section ratio was 1.98.

The other two types of electrodes used in this study were standard round electrodes: ∅4 mm × 450 mm. Both were standard E Fe8 electrodes produced by Elektrode Jesenice d.o.o., Jasenice, Slovenia, the only difference being the outer diameter of the coating. One set had the standard diameter of ∅7.85 mm, and the other set a reduced diameter of ∅6.90 mm (reduced by turning). As the rectangular electrodes had the same coating as the standard round electrodes, all three types of electrodes were the same chemically. The dimensions of the cross-section and ratio coating/metal core are presented in Table 1.

**Figure 4:**
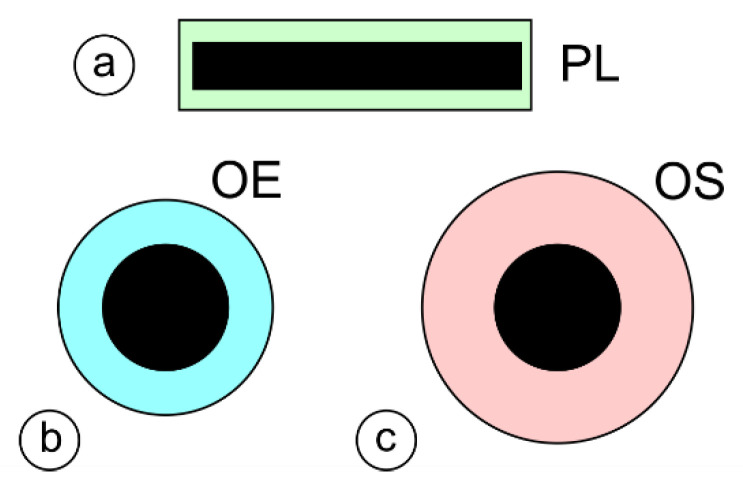
Cross-sections of stick coated electrodes: (**a**) rectangular electrode (PL); (**b**) round standard electrode with reduced outer diameter (OE); and (**c**) round standard electrode.

The pictograms used for the designation of electrode types used in some of the following figures are shown in Figure 4.

### 2.2. Welding of Hardfacing Welds

Bead-on-plate welds were deposited on 250 × 100 × 10 mm EN 10025 [47] S355 JR test plates in the rolling direction, position PA, at room temperature. Before hardfacing, the test plates were sandblasted to clean the surfaces. Welding was performed manually; the tilt angle of the electrode was approximately 20° forward. The polarity was always DC–. The welding current was 120 A for the rectangular electrodes and 160 A for both round electrodes. The welding machine Daihen-Varstroj VARMIG 271-i LCD was used (Daihen Varstroj, Lendava, Slovenia). During welding, the arc length was always kept as short as possible. No preheating was applied, but the maximum interpass temperature was limited to 330 °C.

The heat input was calculated according to EN ISO 1011-1 [48], Equation (1), and technical note PD ISO/TR 18491 [49], where *Q* is the heat input, *k* is the thermal efficiency of the welding process (*k_SMAW_* = 0.8), *U* is the arc voltage, measured as close as possible to the arc, *I* is the welding current in amperes, and *v* is the welding speed.

(1) $$Q=k\frac{U\times I}{v}\times 10^{-3}[\mathrm{kJ}/\mathrm{mm}]$$

**Figure 5:**
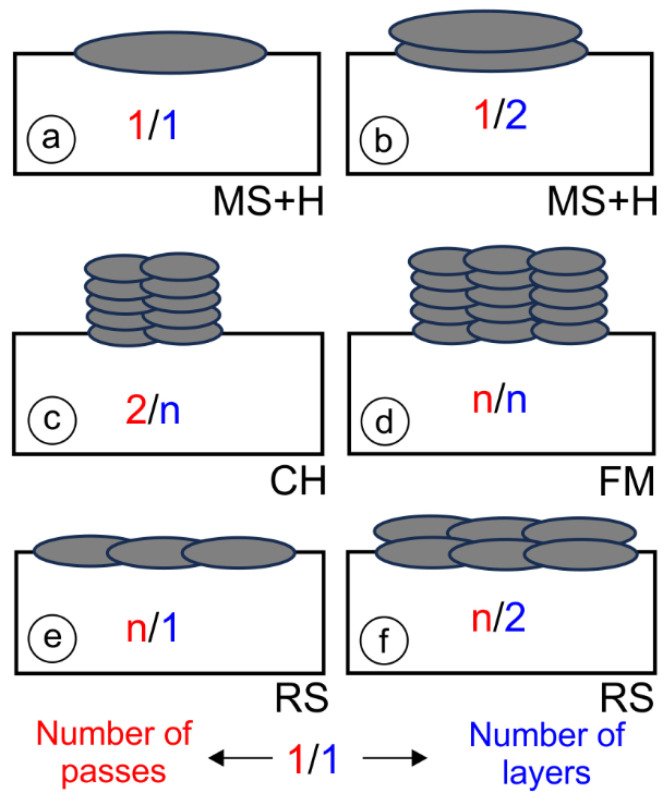
Geometry of hardfacings: (**a**) one-layer and (**b**) two-layer welds, one pass wide; (**c**) multi-layer welds, two passes wide; (**d**) multi-layer welds, several passes wide; (**e**) one-layer weld, several passes wide; and (**f**) two-layer welds, several passes wide.

The welding parameters for all samples with one-layer (PL1, OE1, and OS1), two-layer (PL2, OE2, and OS2), and multi-layer welds are summarized in Table 2. The one-layer, two-layer, and multi-layer welds (up to 30 layers) are shown schematically in Figure 5.

Welds one or two layers thick and one welding pass wide were used for microstructural examinations, the determination of dilution rates (MSs), and hardness measurements (Hs) (Figure 5a,b). The multi-layer welds in Figure 5c,d were used for instrumented Charpy impact tests (CHs) and for fracture mechanics tests (FMs). The samples in Figure 5e,f were used for residual stress measurements (RSs).

### 2.3. Microstructural Examinations and Fractography

The samples for the microstructural examinations were cut from the welds and grinded with P80-P2500 abrasive papers. Etching was performed with 3% Nital etchant, with an etching time of 10 s, according to [49,50]. A Nikon Epiphot 300 optical microscope (Nikon, Tokyo, Japan) equipped with an Olympus DP-12 digital camera (Olympus, Boston, MA, USA) was used to examine the microstructure. The base material, HAZ, and weld metals were examined and analyzed on one- and two-layer welds.

A light microscope, Leica Wild M10 (Leica Mycrosystems, Wetzer, Germany), and FEI Quanta 3D-scanning electron microscope (FEI Company, Hillsboro, OR, USA; now Thermo Fisher Scientific, Waltham, MA, USA) were used for the fractography and crack length measurements of the Charpy and fracture mechanics SENB specimens.

### 2.4. Analyses of Welds and Their Dilution Rates

**Figure 6:**
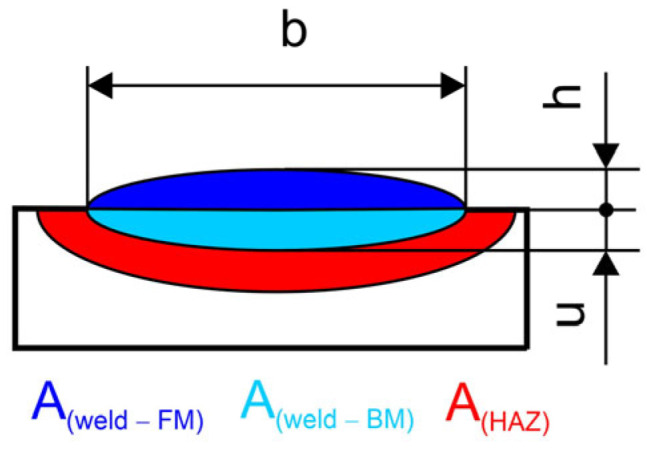
Principle of dilution of the base and filler metals in hardfacing alloys.

In cladding applications, the dilution must be kept as limited as possible to maintain the original filler material composition, and thereby to ensure that the cladding retains its wear or corrosion resistance [51]. The concept of dilution is shown schematically in Figure 6. The dilution can be calculated by using Equation (2). The meaning of the symbols in the equation is explained in Figure 6.

(2) $$Dilution\;rate\;X=\frac{A_{\left(weld-BM\right)}}{A_{\left(weld-BM\right)}+A_{\left(weld-FM\right]}}$$

The geometric dilution is a function of deposit height and depth, while the chemical dilution is related to the enrichment of the elements diffusing from the substrate toward the deposit. Particularly in the case of hardfacing alloys containing a high amount of alloying element, controlling chemical dilution represents a key factor for realizing coatings with high tribological properties through their volume. In fact, a minimum dilution is necessary to guarantee good metallurgical bonding between the coating and the substrate [52]. Samples OS1, OS2, OE1, OE2, PL1, and PL2 were prepared metallographically and measured according to Figure 6. 

### 2.5. Hardness Measurements

**Figure 7:**
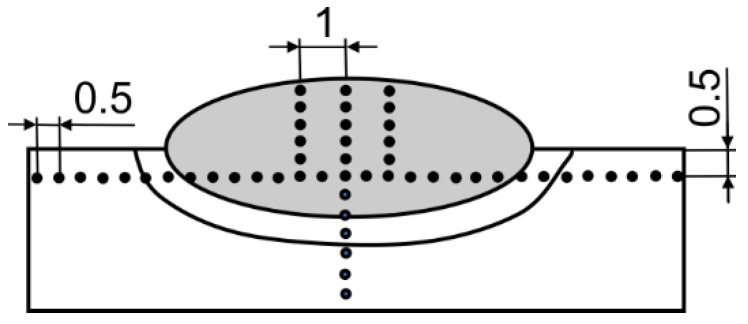
Plan for hardness measurements.

Roell-Zwick Z600 (Zwick Roell Group, Ulm, Germany) was used to measure the Vickers hardness according to EN ISO 9015-1 [53]. A 10 kg load was used for the hardness measurements for 10–15 s. The HV10 hardness was measured in one of three vertical lines from the top of the weld in a direction toward the base material. The measurement points were at a distance of 0.5 mm in the vertical and horizontal directions; see the plan of hardness measurements in Figure 7. Only the welds on PL1 and PL2 specimens were also measured on the horizontal line in the depth x = 0 mm below the base metal surface, because, due to very shallow penetration, at a depth of 0.5 mm there was no weld metal.

### 2.6. Instrumented Charpy Impact Tests

The instrumented Charpy test differs from the conventional Charpy test by measuring the force on the pendulum throughout the test, as well as the absorbed energy. The instrumented Charpy pendulum enables the separation of the total energy (*E_t_*) into crack initiation energy (*E_i_*) and crack propagation energy (*E_p_*), and the result is the possibility of a more accurate assessment the material’s toughness. 

**Figure 8:**
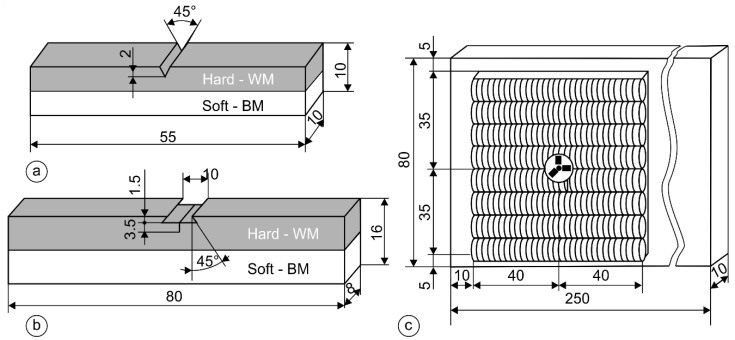
Geometry of samples for different tests: (**a**) Charpy specimen; (**b**) SENB specimen; and (**c**) sample for residual stress measurements with a stain gauge rosette.

For the Charpy impact tests, an instrumented Charpy pendulum Amsler RPK300 (Amsler Prufsysteme, Neftenbach, Switzerland) was used, with a data acquisition rate of four mega samples reading per second, in accordance with ISO 148-1 [54]. The geometry of the Charpy specimens (Figure 8a) was the standardized 10 × 10 × 55 mm, with a 2 mm deep ISO V-shaped notch. The position of the ISO V-notch was from the side of the weld metal toward the base material in the thickness direction. All the instrumented Charpy tests were performed at room temperature (20 °C).

### 2.7. Fracture Mechanics Tests

The fracture mechanical tests were carried out in accordance with the ASTM E1820 Standard [55]. Single Edge Notch Bend (SENB) specimens were used in this study, where the geometry of the specimens is shown in Figure 8b. The dimensions of the specimens were 16 × 8 × 80 mm, with a notch made by the EDM—erosion process. The pre-fatigue and post-fatigue sequences of the tests were carried out on an RUMUL 160 Nm Cracktronic machine (Rumul, Schaffhausen, Switzerland). The SENB test was carried out on a 10 kN Smitweld TTU 2002 (Smitweld b.v., now Lincoln Smitweld b.v., Nijmegen, The Netherlands). Three specimens were produced, all with a notch in the welded layer. For the evaluation of the fracture mechanics tests, the normalization method was used in accordance with the ASTM E1820 Standard.

### 2.8. Residual Stress Measurements

**Figure 9:**
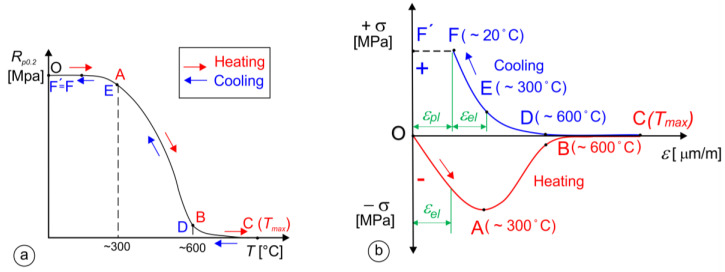
Residual stress in construction steels: (**a**) change in *R_p_*_02_ with temperature *T*; (**b**) weld deformation cycle.

During the SMAW process, part of the base material and the filler material melt, and the nearby area (HAZ) of the fusion zone heats up and then cools down rapidly. Due to the surrounding cool base material, deformations are hindered, which leads to the appearance of residual stresses. Figure 9a shows the variation in yield stress *R_p_*_0.2_ with temperature schematically. For non-alloyed structural steels, *R_p_*_0.2_ starts to decrease above 300 °C, and at about 600 °C, it is already significantly lower than at room temperature. Figure 9b shows the deformation cycle for the welding of non-alloyed structural steel. In the heating process, compressive stresses are generated and decrease with increasing the temperature as a result of decreasing *R_p_*_0.2_, and, in the cooling process, tensile stresses increase with decreasing the temperature, as a result of increasing the *R_p_*_0.2_ of the base material. At room temperature, due to the limited possibility of expansion and contraction of the cool base material, residual stresses appear in the material. Residual stress measurements were performed by the standardized hole-drilling method (ASTM E837) [52]. Hottinger three-elemental strain gauge RY061 rosettes were used for the measurements. The Vishay measurement system RS200 (Vishay Measurements Group GmbH, Heilbronn, Germany) was used for the residual stress measurements. The geometry of the two-layer hardfacing weld specimen is presented in Figure 8c, where the residual stress measurement point is in the middle of the surface. Two different hardfacing welds were measured. The first hardfacing weld was welded by rectangular stick electrodes (∅1 × 12.56 mm) and the second was welded with round electrodes (∅4/∅7.85). 

## 3. Results and Discussion

### 3.1. Analysis of Arc Burning by High-Speed Camera

**Figure 10:**
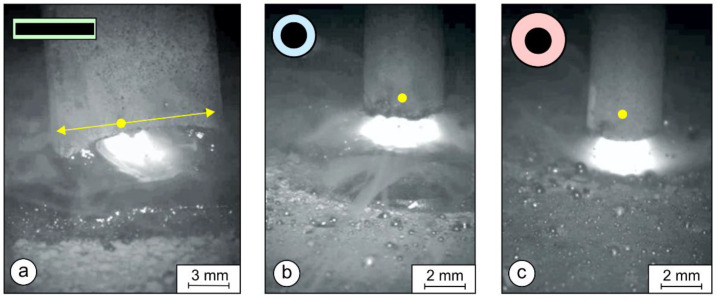
SMAW arc taken by high-speed camera: (**a**) rectangular stick electrode (∅1 × 12.56 mm); the arc was traveling from one side to the other and back (yellow arrows); (**b**) thinned round electrode (∅4/∅6.90 mm); and (**c**) conventional round-shaped electrode (∅4/∅7.85 mm); the arc stays at the same position (yellow points).

Arc burning was analyzed by a high-speed camera: Cavitar C300 (Cavitar Ltd., Tampere, Finland). In the case of the rectangular stick electrode, the arc was traveling all the time from one side to the other and back (yellow arrows in Figure 10a). Therefore, the local heat input decreased and, consequently, the penetration depth decreased. The arc length was approximately 2.8–3.1 mm. In the case of the round electrodes in Figure 10b,c, the arc stays at the same position (yellow points in both subfigures). This increases the local heat input and, consequently enlarges the penetration depth. The length of the arc with the round electrodes was approximately 2–2.5 mm.

The voltage was higher for the rectangular electrode than for the round ones. The conductive cross-section was smaller than the round electrode, where the difference in volume and composition of the coatings may have led to a disparity, similar to that explained by Crespo [40] for a tubular electrode.

The frequency of the arc traveling from one side of the electrode to the other, determined from the high-speed-camera videos, was 3.5–4.5 journeys per second.

**Figure 11:**
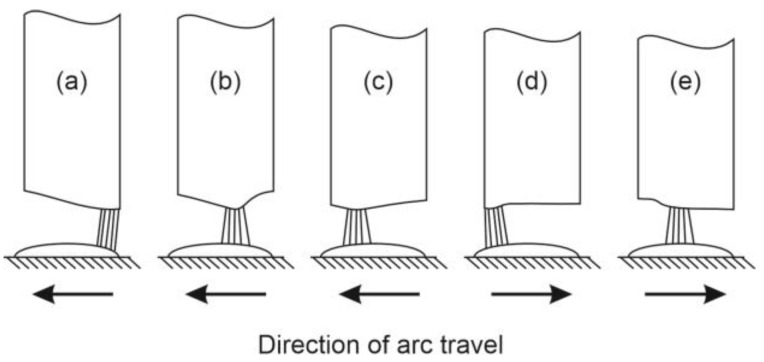
(**a**) The arc burns where the distance to weld pool is the shortest; (**b**–**d**) as the electrode melts, in the search for the shortest distance to the weld pool, the arc traveles from one edge of the electrode to the other; (**e**) then the journey back starts; the frequency of the journeys, approx. 3.5–4.5-times per second, was determined from the high-speed-camera videos.

Commuting of the arc can be explained as follows: The electric resistance of the arc increases with its length. This means that the arc continuously searches for the shortest distance between the electrode and the weld pool. If, at a certain moment, the shortest distance is on the right edge of the rectangular electrode, the arc will be located on the right edge (Figure 11a).

**Figure 12:**
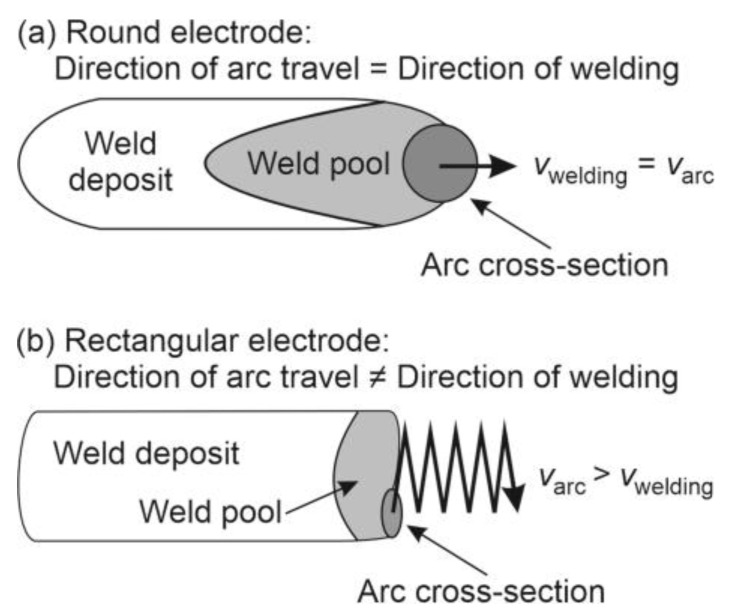
Welding speed and arc-travel speed: (**a**) in the case of the round electrode; (**b**) in the case of the rectangular electrode.

As the area in contact with the arc melts, this side of the electrode soon becomes shorter than the adjacent areas further to the left. Therefore, the arc starts to travel to the left, to the area where the distance to the weld is now shorter (Figure 11b,c,e). When the left edge of the electrode melts away, the arc travels back to the right edge. Due to commuting, the velocity of arc-travel is composed of two components: welding speed and arc-commuting speed. Therefore, the actual velocity of the arc is notably higher than the welding speed, and the distance traveled by the arc is notably longer than the weld deposit (Figure 12). Considering the width of the rectangular metallic core and the frequency of commuting determined from the high-speed-camera videos, the actual arc-travel speed was approx. 40–50 mm/s.

However, despite the high actual arc-travel speed, due to the lower welding speed, the heat inputs per unit length of the deposit with rectangular electrodes were higher than with round ones (Table 2). Therefore, due to the higher heat inputs per unit of deposit length with rectangular electrodes, one could jump to the conclusion that, in spite of the higher actual arc-traveling speed, the penetration should be deeper and dilution rates higher. However, considering only heat inputs per unit of deposit length is misleading because the decisive influence comes from elsewhere. When welding with a round electrode without weaving, the point of heat input always stays at the mid-width of the weld bead. Consequently, the temperature in the middle is high, solidification rates in the mid-width are low, and the weld pool is long. Hence, the base metal in the middle is exposed to very high temperatures for a long time, which results in deep penetration and, therefore, high dilution rates, but this is not the case for the rectangular electrodes if they are wide enough. In such a case, the width of the arc is small compared to the electrode width (Figure 10a), and the arc is travels. Due to arc travel, not only does the actual arc-traveling speed increase, but the point of heat input also moves from left to right and back, where the heat is distributed evenly over the entire bead width. Consequently, the melt temperature remains lower, the melt pool is shorter, and the penetration at the mid-width remains as shallow as at the edges. In this way, less base metal melts and the dilution rates remain lower.

### 3.2. Dilution Rate

**Figure 13:**
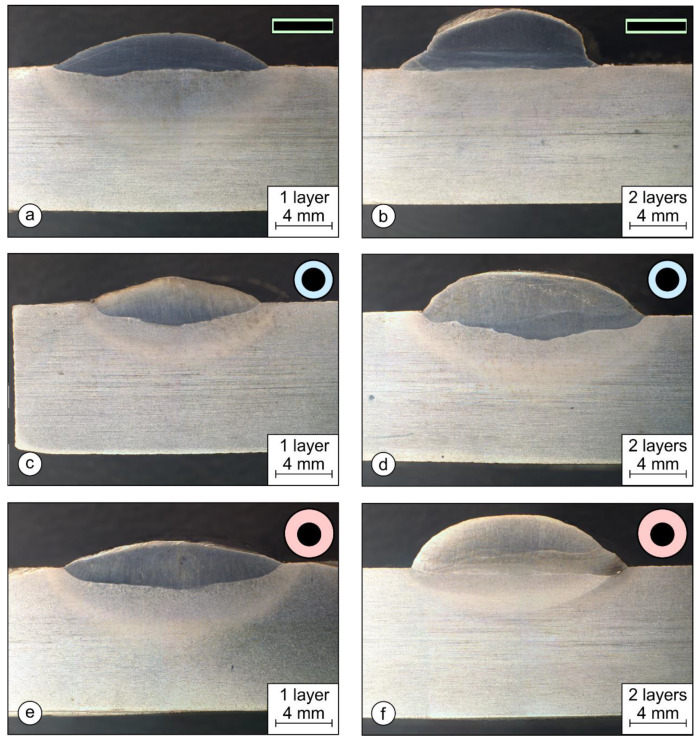
Macro-sections of hardfacing welds: (**a**,**b**) represent the rectangular coated stick electrode; (**c**,**d**) represent the round thin-coated electrode (∅4/∅6.90 mm); and (**e**,**f**) represent the conventional round coated electrode (∅4/∅7.85 mm).

Figure 13 shows the cross-sections of welds for all three types of coated electrodes made with one or two layers. The dimensions of the welds are summarized in Table 3. Figure 13a,b show the macro-sections of welds PL1 and PL2 made by a rectangular stick electrode with one- (PL1) and two-layer (PL2) welds. In both pictures, very shallow penetration is visible, which is directly related to the dilution rates. The PL2 sample had the absolute lowest dilution rate among all the samples with *X* = 6.8%. 

Among the two-layered samples, the dilution rate for PL2 was 2.48-times lower than that of OS2 and 4.83-times lower than that of OE2 (Table 3). Similarly, sample PL1 had the lowest dilution rate of all the single-layer samples with a value of *X* = 15.9%, 2.86-times less than OE1 and 3.25-times less than OS1. The macro-sections of samples OE1 and OE2 are shown in Figure 13c,d, and the macro-sections of samples OS1 and OS2 are shown in Figure 13e,f.

### 3.3. Microstructural Analysis

**Figure 14:**
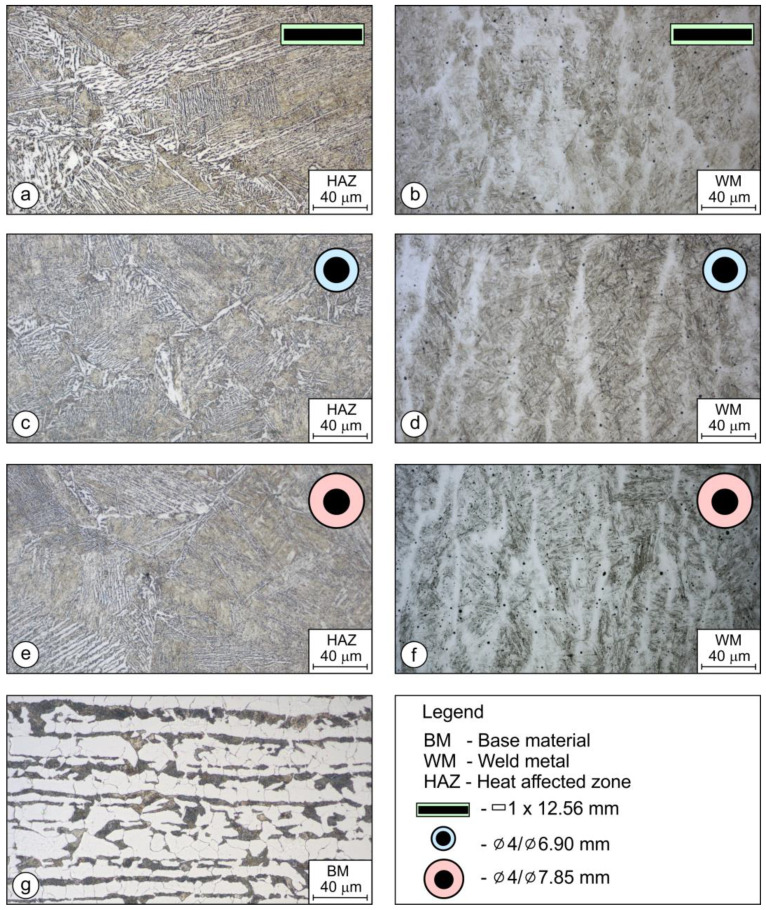
Microstructure of hardfacing welds: (**a**,**b**) HAZ and WM welded by the rectangular coated stick electrode; (**c**,**d**) HAZ and WM welded by the round thin-coated electrode (∅4/∅6.90 mm); (**e**,**f**) HAZ and WM welded by the standard round coated electrode (∅4/∅7.85 mm); and (**g**) base material.

The microstructures of HAZ’s of the deposited welds can be observed in Figure 14a–e. In all three variants, as a result of the high cooling rates, the microstructure consists of martensite and bainite. In the case of Figure 14a, which shows the HAZ of the weld deposited with the rectangular stick electrode, we can observe an increased content of Widmanstaetten ferrite, which also has a positive influence on higher impact toughness in comparison to the higher content of martensite, which appeared in the HAZs of hardfacing welds welded by both round electrodes (∅4/∅6.90 mm and ∅4/∅7.85 mm). The microstructure of the weld metal can be seen in Figure 14b,d,f. The microstructure is martensitic, due to the higher contents of carbon and alloying elements. The weld metal microstructure deposited with the rectangular electrode is shown in Figure 14b. The microstructure consists of martensite, which has a very fine appearance of laths. A much coarser microstructure of the weld can be observed in the weld deposited with conventional electrodes (Figure 14d,f). The coarse microstructure is related to the point-oriented heat input when welding with the electrodes that have round cross-sections. Figure 14g shows the microstructure of the base material, which consists of ferrite and perlite.

### 3.4. Hardness Measurements

**Figure 15:**
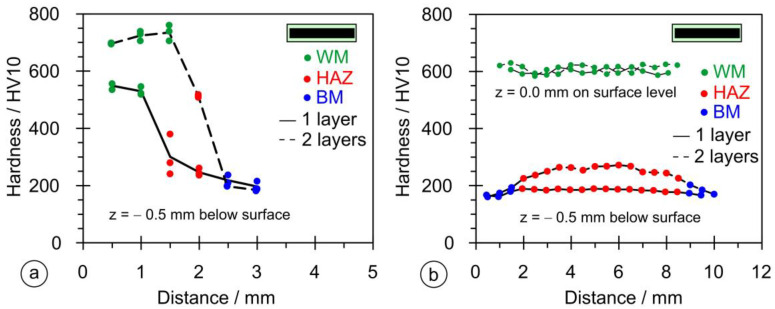
Results of the hardness measurements on welds made with the rectangular stick electrode (∅1 × 12.56 mm): (**a**) in the vertical direction; (**b**) in the horizontal direction.

As stated in Section 2.5, the dimensional measurements were performed on six samples in both vertical and horizontal directions. Figure 15a shows the results of the hardness measurements in the vertical direction for the single- and double-layer hardfacing welds for samples PL1 and PL2. The single-layer hardfacing weld has a maximum hardness < 600 HV, while the double-layer weld has a maximum hardness above 700 HV. The rate of hardness decreases across the cross-section and has a similar slope for the single- and double-layer hardfacing welds. Figure 15b shows the results of the hardness measurement in the horizontal direction; at the measuring point 0.5 mm below the surface, the hardness does not exceed 300 HV due to lower penetration (only the lower part of the HAZ region was reached), while at the surface of the base material, it rises to a value of 600 HV (weld metal region).

**Figure 16:**
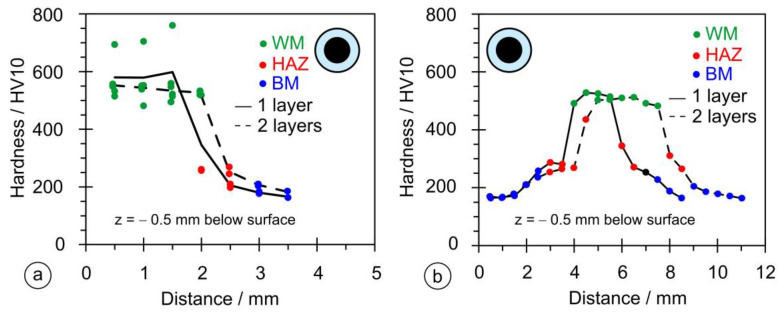
Results of hardness measurements on welds made with the thin-coated electrode (∅4/∅6.90 mm): (**a**) in the vertical direction; (**b**) in the horizontal direction.

Figure 16 shows the results of the hardness measurements in both directions for samples OE1 and OE2. Compared to the rectangular stick electrode, the OE type shows a larger scatter of measured values (Figure 16a); the average hardness in the vertical direction does not exceed 650 HV and is higher for the one-layer hardfacing weld. In the horizontal direction, Figure 16b, the hardness values exceed 500 HV, indicating a deep penetration depth and significant dilution rate.

**Figure 17:**
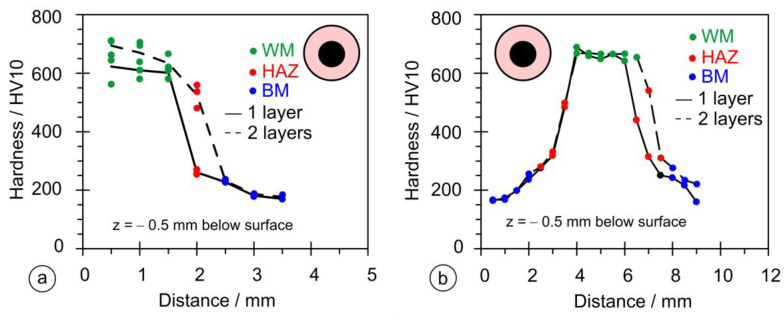
Results of the hardness measurements on welds made with the conventional coated electrode (∅4/∅7.85 mm): (**a**) in the vertical direction; (**b**) in the horizontal direction.

Figure 17 shows the hardness measurements for samples OS1 and OS2. The measurements in the vertical direction, Figure 17a, produced a maximum hardness higher than for the OE series and lower than for PL2, while the hardnesses measured horizontally are the absolute highest of the three series, Figure 17b, due to higher alloying because of the higher thickness of the coating.

Increasing hardness generally improves resistance to abrasive wear. Nevertheless, to evaluate the improvement, wear tests are inevitable. Therefore, pin-on-plate abrasive wear tests are being carried out at present. As numerous combinations of electrode-type/layer numbers must be tested, testing is still ongoing, and only some preliminary results are available at the moment. Nevertheless, it can already be said that the improvement of hardfacings’ performance by using rectangular electrodes is significant. The maximum improvement was measured with two-layer hardfacings, where, if welded with a rectangular electrode, the material loss was only 64% of that measured for specimens welded with round electrodes that have the same metal core/coating ratio.

### 3.5. Instrumented Charpy Tests

**Figure 18:**
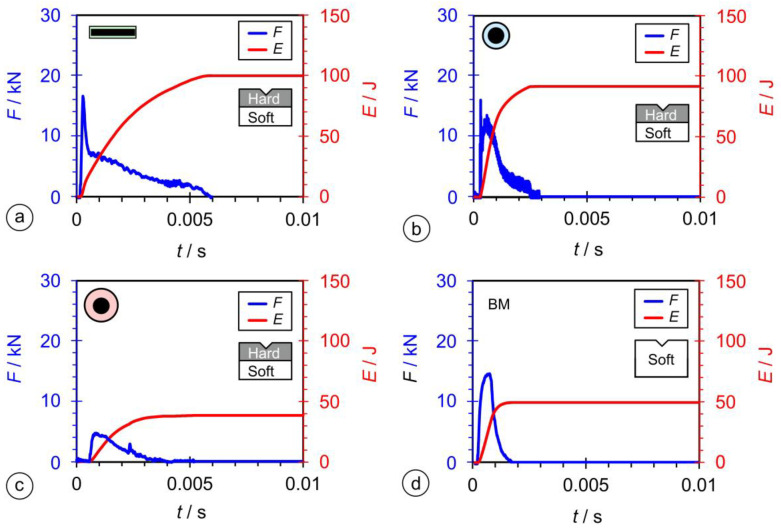
Results of instrumented Charpy tests: (**a**) rectangular stick electrode (∅1 × 12.56 mm); (**b**) thinned round electrode (∅4/∅6.90 mm); (**c**) conventional round electrode (∅4/∅7.85 mm); and (**d**) base material.

Typically, the results of an instrumented Charpy test are described by two curves: *F* versus *t* (blue curve) and *E* versus *t* (red curve). Impact toughness tests were carried out on all three hardfacing welds welded by all three types of electrodes (PL-CH, OE-CH, and OS-CH samples), and a test on the base material S355 J2 was added for comparison. The highest absorbed energy (close to 100 J) was measured for the PL-CH sample (Figure 18a). In the case of the OE-CH sample, the absorbed energy was about 10% lower (Figure 18b). In the case of the OS-CH sample, the absorbed energy was the lowest (Figure 18c). The energy absorbed in the base material was around 50 J (Figure 18d).

The *F* versus *t* curve (blue) provides an indication of the force required to initiate the crack up to the maximum force, while the remainder of the curve represents the energy required to propagate the crack. The maximum crack initiation force was measured for the PL-CH specimen, Figure 18a, and the minimum for the OE-CH specimen. Similarly, the maximum crack propagation energy was measured for the PL-CH specimen, Figure 18a, and the minimum for the OS-CH specimen (Figure 18c). The force for OM was lower than for the PL-CH specimen and OE-CH, and higher than for the OS-CH specimen (Figure 18d).

### 3.6. Fracture Mechanics Tests

For homogeneous brittle materials, the ASTM E399 Standard [56] is usually used, while in the case of inhomogeneous and ductile materials, the ASTM E1820 Standard [55] is more appropriate. ASTM E1820 is also more appropriate for materials in which the crack propagation passes through regions with different mechanical properties, such as the hardfacing weld metal, HAZ, and base material. 

**Figure 19:**
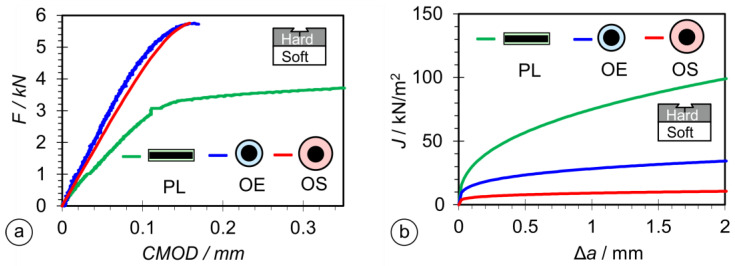
Results of fracture mechanics tests: (**a**) force-*CMOD* diagram; (**b**) resistance curves *J*-Δ*a*.

Figure 19a shows the *F*-*CTOD* curves (*CTOD* = crack mouth-opening displacement) for specimens PL-FM, OE-FM, and OS-FM, where all have notches in the hardfacing welds. The *F*-*CTOD* curves for specimens OE-FM and OS-FM (welded by round electrodes) were very similar in shape and value. They reached values of approximately 5.5 kN, with a CMOD value of about 0.15 mm, whereas the value obtained for the PL-FM specimen (welded by the rectangular stick electrode) was considerably lower, with a much higher CTOD of about 0.3 mm.

The values of the resistance curve, *J*-integral, are shown in Figure 19b. The highest value was achieved by the PL-CH sample, while significantly lower values were achieved by the OE-FM and OS-FM samples (welded by round electrodes).

**Figure 20:**
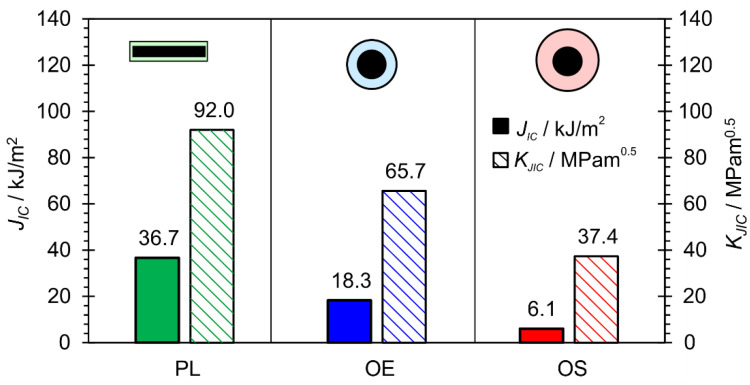
Results of fracture mechanics tests: critical *J_IC_* an *K_JIC_*.

The *J_I_*c and *K_JIC_* values are shown in Figure 20. The PL-FM sample had the highest *K_JIC_* value, while the OE-FM and OS-FM samples had lower values. The *J_IC_* value of the PL-FM specimen was the highest, while the value of the OS-FM specimen was the lowest.

**Figure 21:**
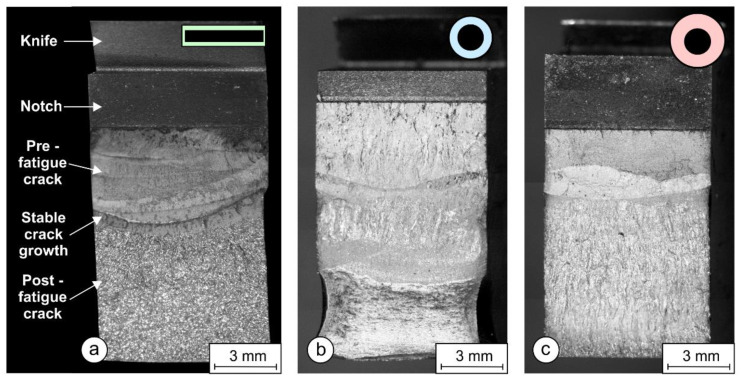
Fractured surfaces of weld metal welded by: (**a**) a rectangular stick electrode (∅1 × 12.56 mm); (**b**) a thinned round electrode (∅4/∅6.90 mm); (**c**) a conventional round electrode (∅4/∅7.85 mm).

Figure 21 shows the fracture surfaces of the PL-FM, OE-FM, and OS-FM samples with the characteristic areas marked. At the top is a knife for the CMOD sensor attachment, followed by a mechanical notch. This is followed by the pre-fatigue crack area, which is actually the preparation stage for SENB fracture mechanics tests. The next area is the stable crack growth area, which is somehow the result of the SENB test. On the fracture surface areas, the initial crack length, *a*_0_ (the start of stable fatigue growth), and physical crack length, *a_p_* (the end of stable crack growth), are measured in nine points, according to the ASTM E1820 Standard procedure. The final area is the post-fatigue marking area.

**Figure 22:**
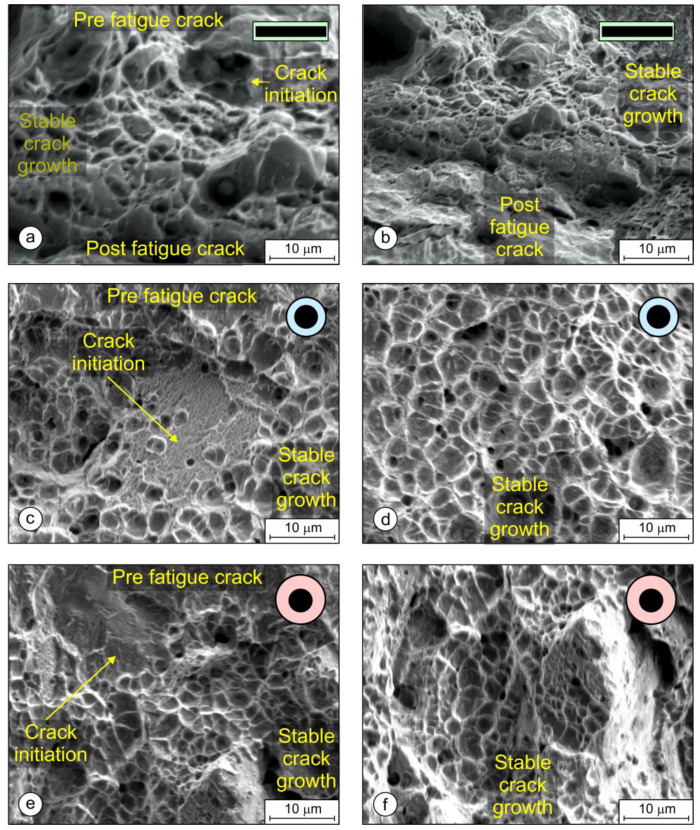
Fracture surfaces of weld metal from SEM in the area of crack incitation and stable crack growth: (**a**,**b**) rectangular stick electrode (∅1 × 12.56 mm); (**c**,**d**) thinned round electrode (∅4/∅6.90 mm); and (**e**,**f**) conventional round electrode (∅4/∅7.85 mm).

The fracture surfaces were examined with an SEM electron microscope. Figure 22 shows the areas of pre-fatigue, the crack initiation site, and the stable crack growth beneath. The left images (Figure 22a,c,e) were taken on the initial crack front where the crack initiated and stable crack growth began. The right images (Figure 22b,d,f) were taken a little below the crack initiation area, where the crack propagated in a stable manner. To facilitate a comparison between the samples, all the images were taken at the same magnification. Figure 22a,b shows areas with deep dimples on the PL-FM sample, which correspond to a tough fracture. Figure 22c,d show the fracture surface of the OE-FM sample, where less deep dimpled areas can be seen, and, occasionally, some small brittle areas appear. Finally, Figure 22e,f show fracture surfaces for the OS-FM sample, where most of the brittle areas can be seen, which influenced decreasing the *K_JIC_* value and the lower resistance curve.

The impact and fracture toughness of a material is influenced by the quality and size of the microstructural constituents. In most cases, a higher hardness of the microstructural constituents results in lower toughness, and a larger crystal grain also results in lower toughness. The lower local heat input when welding with a 1 × 12.56 mm rectangular electrode resulted in a higher cooling rate and, hence, finer martensitic laths with slightly higher hardness were obtained in the hardfacing. From the microstructures of the hardfacings in Figure 14b,d,f, it can be seen that the width of the martensitic crystal grains is the smallest when welding with a 1 × 12.56 mm flat-stick electrode, on average, 20–30 μm, while, in the case of the round electrodes (∅4/∅6.90 mm and ∅4/∅7.85 mm) it is, on average, about 35 μm or more. Due to the finer structure, the impact toughness of hardfacings made with the rectangular electrode is the highest (Figure 18). A similar effect of the microstructure was also observed in the SENB tests and on the fracture surfaces of the SENB specimens cut from hardfacings. In the case of the hardfacing welded with the 1 × 12.56 mm rectangular electrode, smaller and more numerous dimples can be observed (average size: 2–3 μm; Figure 22b) than in the case of the round electrodes (5 μm and 3–4 μm; Figure 22d and Figure 22f, respectively), which also indicates a higher resistance of the material to crack propagation during the SENB test. This, in turn, had a direct influence on the K_JIC_ and the higher resistance curve of the hardfacing welded with the rectangular electrode (Figure 19b).

### 3.7. Residual Stress

Residual stresses are stresses that remain in the body after the action of external forces has stopped. They were measured by the incremental hole-drilling method, according to the ASTM E 837 Standard [52]. Specimens PL-RS and OS-RS with two-layer hardfacings were used. 

**Figure 23:**
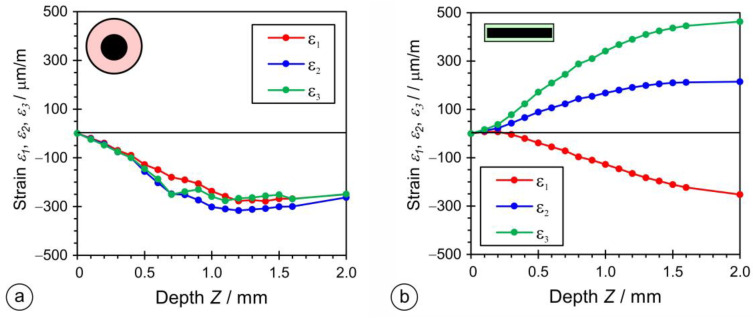
Results of the residual stress measurements. Strains measured with a three-elemental strain-gauge rosette on hardfacings welded by: (**a**) a rectangular stick electrode (∅1 × 12.56 mm); (**b**) a conventional round electrode (∅4/∅7.85 mm).

The deformations were measured in three-elemental strain-gauge rosettes in three directions, with an offset angle of 45°. Figure 23 shows the results of the strain measurements with increments of 0.1 mm (after each incremental drilling steep). For the OS-RS specimen, the strain values of all three directions, *ε*_1_, *ε*_2_, and *ε*_3_, were negative. In the case of the PL-RS sample, strain values *ε*_1_, and *ε*_2_ were positive and *ε*_3_ was negative. A residual stress measurement was not performed for the OE-CH sample.

**Figure 24:**
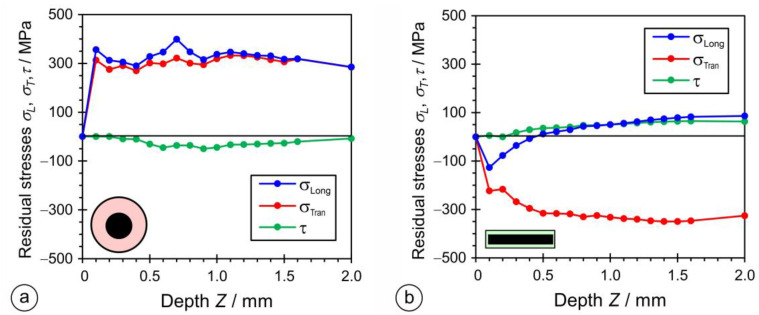
Residual stresses in hardfacings welded by: (**a**) a rectangular stick electrode (∅1 × 12.56 mm); (**b**) a conventional round electrode (∅4/∅7.85 mm).

The principal residual stresses and angle to principal stresses were calculated from the measured values of the strains by using the ASTM E837 Standard procedure. The principal longitudinal (*σ_Long_*) and transversal (*σ_Tran_*) and shear (*τ*) residual stresses were calculated by using the Mohr theory. The plots in Figure 24 show that residual stresses *σ_Long_* and *σ_Tran_* are positive for the OS-RS sample, with values around 300 MPa throughout the thickness, while the shear residual stress, *τ*, is slightly negative.

For the PL-RS sample, the residual shear stress (*τ*) was slightly positive and reached values between 0 and 100 MPa, *σ_Long_* was negative up to a depth of 0.5 mm, and then followed the values of shear residual stresses, *τ*, while transversal residual stresses (*σ_Tran_*) remained negative, and reached maximum negative values of around −310 MPa.

## 4. Conclusions

A new type of coated electrode with a rectangular cross-section of the metallic core (∅1 × 12.56 mm) was developed and tested successfully. The coating was identical to the commercially available electrode E DUR 60 R (EN 14 700, E Fe 8). The hardfacing test welds were deposited on S355 J2 structural steel. 

Two standard round electrodes (∅4 mm) and coating diameters of ∅6.90 and ∅7.85 mm were used for comparison. Commuting of the arc along the width of the metal core was observed in the case of the rectangular electrode (Figure 10a).

The dilution rate, *X*, for the rectangular electrode was significantly lower than for round ones (Table 3). 

Metallographic examination revealed that the content of Widmanstaetten ferrite was the highest in the HAZ of welds made with rectangular electrodes, a bit lower with the thin-coated round electrode, and the lowest with the thick-coated round electrode (Figure 14). The microstructures of the weld metal were martensitic–bainitic for all the electrodes. But, the bainite content of the rectangular electrode was higher than the round electrodes, which was reflected in better toughness values. Carbides of a spherical shape were observed in the microstructures, their size and fraction being the highest with the thick-coated round electrode (Figure 14), which was the consequence of the highest ratio of non-metallic coating to the metallic core. 

The absolute maximum measured hardness of approx. 751 HV10 was measured in the vertical direction in two-layer hardfacings made with the rectangular electrode. The highest scattering of hardness was observed with the thin-coated round electrodes. 

The *F*-*t* and *E*-*t* curves were measured with the Charpy instrumented test. The highest absorbed impact energy of 100 J was measured for the specimens welded with the rectangular electrode (Figure 18). 

*J_IC_* and *K_JIC_* values of hardfacings (Figure 20) were determined with fracture mechanical tests. Both values were the highest with the rectangular electrode. Also, SEM microscopy confirmed that the characteristics of fracture surfaces were more ductile in hardfacings made with the rectangular electrode and more brittle with the round electrodes (Figure 22). 

The residual stresses were determined with the incremental drilling method on samples cut from two-layer hardfacings. In hardfacings made with rectangular electrodes, the residual stresses were between −310 MPa and +80 MPa, while, with round electrodes, they were between −53 MPa and +411 MPa.

## Figures and Tables

**Table 1:** Dimensions, cross-sections, and ratio of coating/metal core for OS, OE, and PL series electrodes.

Type of Electrode	Dimension/mm		Cross-Section/mm^2^		Ratio
	Metal Core	Coating	Metal Core	Coating	Coating/Metal Core
PL-series	∅1 × 12.56	∅2.80 × 13.35	12.56	24.82	1.98
OE-series	∅4.00	∅4/∅6.90	12.56	24.81	1.98
OS-series	∅4.00	∅4/∅7.85	12.56	35.81	2.85

**Table 2:** Welding parameters and purpose of specimens.

Weld	I/A	U/V	T/s	Tstart/°C	Q/kJ mm^−1^	Weld Type ^1^	Purpose
PL1	120	23.5	33.8	20	0.89	1/1	Microstructural analysis + Dilution rates
PL2	120	21.6	38.1	20	1.05	1/2	
OE1	160	20.3	16.5	20	0.61	1/1	
OE2	160	20.2	19.6	20	0.72	1/2	
OS1	160	19.4	17.1	20	0.57	1/1	
OS2	160	20.3	18.6	20	0.64	1/2	
PL-CH	120	29.0	47.0	20	1.01	2/n	Charpy impact tests
OE-CH	160	29.0	21.4	20	0.62	2/n	
OS-CH	160	32.0	21.1	20	0.80	2/n	
PL-FM	120	29.0	48.0	20	1.04	n/n	Fracture mechanics tests
OE-FM	160	29.0	21.2	20	0.66	n/n	
OS-FM	160	32.0	19.4	20	0.71	n/n	
PL-RS	120	21.6	38.1	20	1.05	n/2	Residual stress measurement
OS-RS	160	20.3	18.6	20	0.64	n/2	

^1^ Weld type—see Figure 5.

**Table 3:** Dimensions and areas of surface welds and their dilution rates.

		Specimens					
		PL1	PL2	OE1	OE2	OS1	OS2
*b*	mm	15.0	14.7	14.3	15.0	14.6	14.3
*h*	mm	2.3	4.5	1.9	3.1	1.7	3.4
*u*	mm	0.6	0.4	1.5	1.7	1.7	0.8
*b_HAZ_*	mm	18.7	20.8	17.0	19.4	14.6	17.8
*u_HAZ_*	mm	5.9	5.1	2.4	3.3	3.4	2.7
*u_HAZ-RL_*	mm	6.5	5.5	3.9	5.0	5.1	3.2
*A_Weld_*	mm^2^	28.5	48.6	26.2	48.4	34.5	42.7
*A_Weld-FM_*	mm^2^	24.0	45.3	14.2	32.5	16.6	35.5
*A_Weld-BM_*	mm^2^	4.5	3.3	11.9	15.9	17.9	7.2
*A_HAZ_*	mm^2^	84.1	72.1	34.2	50.2	47.4	32.6
*X*	%	15.9	6.8	45.6	32.9	51.8	16.9

## Data Availability

Data are contained within the article.
